# Safety and efficacy of the mAXIS stapes prosthesis

**DOI:** 10.1007/s00405-024-08854-z

**Published:** 2024-07-28

**Authors:** Nicholas Bevis, Marc A. Hüser, David Oestreicher, Dirk Beutner

**Affiliations:** Department of Otorhinolaryngology, Head and Neck Surgery, Nicholas Bevis, University Medical Center Goettingen, Robert-Koch- Straße 40, 37075 Goettingen, Germany

**Keywords:** Middle-ear prosthesis, Stapedotomy, Stapes surgery, Otosclerosis, mAXIS stapes prosthesis

## Abstract

**Purpose:**

Otosclerosis leads to a fixed stapes footplate and thus to hearing loss. The predominant treatment method is surgery, with various types of stapes prostheses available. The aim of this study was to investigate the safety and efficacy of the new mAXIS Stapes Prosthesis.

**Methods:**

34 cases of otosclerosis were implanted with the new mAXIS Stapes Prosthesis. Comprehensive clinical assessments, including pre- and postoperative pure tone audiometry was performed at short-term (ST) follow-up at 25 (± 15) days and mid-term (MT) follow-up at 181 (± 107) days. The pure tone average of 0.5, 1, 2 and 3 kHz (PTA4) was calculated.

**Results:**

In all cases, the application of the prosthesis was successful and straightforward. The postoperative PTA4 air-bone gap was 10.7 ± 5.2 dB at ST follow-up (*n* = 34) and 8.3 ± 4.1 dB at MT follow-up (*n* = 18). In 61% of cases, the ABG-closure was within 10 dB and in 100% of cases within 20 dB at MT follow-up.

**Conclusion:**

Findings of this study support that the mAXIS Stapes Prosthesis is safe for implantation and shows promising audiological outcome. Future investigations will contribute its long-term efficacy and safety profile.

## Introduction

Modern stapes surgery is a reliable treatment for patients with otosclerosis with excellent postoperative hearing outcome [1]. Since Shea’s first description of the procedure [2], improvements in surgical technique have led to the emergence of alloplastic stapes prostheses [3]. Attaching the prosthesis onto the incus is a crucial step during the surgery. Nowadays, various types of prostheses are available, which differ in the technique of coupling to the long process of the incus, but also in the material.

Various alloplastic materials have proven themselves as prosthesis materials, whereby titanium has proven itself due to its excellent biocompatibility and mechanical properties as well as its magnetic resonance imaging (MRI) compatibility [4–6]. Prostheses also differ in terms of dimensions and particularly in the mechanism by which they are attached to the incus. This is achieved by crimping, clipping or heat activation. Since erosion or necrosis of the incus can occur as a result of too tight or lose crimping, as well as mechanical trauma [7–10], the à Wengen piston prosthesis was introduced in 2000, with no additional “crimping” needed to fix it onto the incus [11]. Its clip design avoids the circular enclosure of the incus from conventional prostheses, reducing the risk of necrosis due to excessive pressure on the incus. However, a review of 22 studies could neither find a difference of the audiological outcome nor a discernible difference in adverse events with any crimping method [12].

The aim of this study was to investigate the intraoperative handling of the new mAXIS Stapes Prosthesis (MED-EL, Innsbruck, Austria) and to record the audiological outcome and possible adverse events during or after implantation.

## Materials and methods

**Fig. 1:**
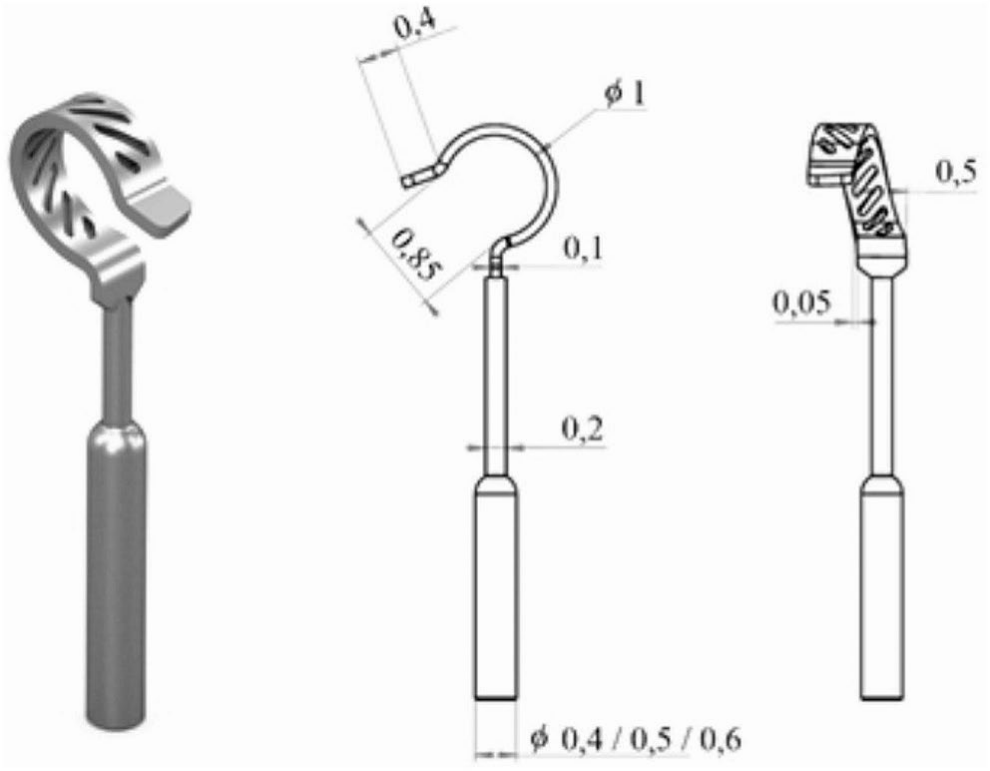
3D rendering of the mAXIS stapes prosthesis with the corresponding diameters in mm

The mAXIS Stapes Prosthesis was developed in collaboration with MED-EL (MED-EL, Innsbruck, Austria). The prosthesis is made of pure titanium using a perforated loop design for coupling the prosthesis onto the long process of the incus. The prosthesis is available with diameters of 0.4, 0.5 and 0.6 mm. The functional length of the prosthesis varies from 3.50 to 5.50 mm in increments of 0.25 mm. The prosthesis is crimped onto the long process of the incus and has a 0.5 mm wide and 0.1 mm thick, perforated loop to reduce the pressure surface direct onto the mucosa. The shaft allows bending to adapt the implant for intraoperative needs. The perforated wider loop is a good compromise reducing the pressure onto the mucosal tissue surface and the rebound effect because of the resistance of titanium during crimping. To ensure a stable anchorage to the prosthesis loop, another advantage could be mucosal overgrowth through the perforated loop, leading to a better perfusion of the mucosa of the incus and maintain nutrition of the lenticular process. (Fig. 1)

The study was conducted in a prospective, monocentric setting and took place between January 2021 and November 2023.

Data collection included detailed audiometric assessment preoperative, at short-term (ST) follow-up after 25 (± 15) days and at mid-term (MT) follow-up after 181 (± 107) days. 34 cases were present at the preoperative and ST follow-up and 18 at MT follow-up. Additionally, postoperative complications were gathered. A standard audiometer (AT1000, Auritec GmbH, Hamburg, Germany) was employed for audiological assessment. The Pure Tone Average (PTA4) at frequencies 0.5, 1, 2, and 3 kHz was computed following the guidelines of the American Academy of Otolaryngology-Head and Neck Surgery [13].

**Fig. 2:**
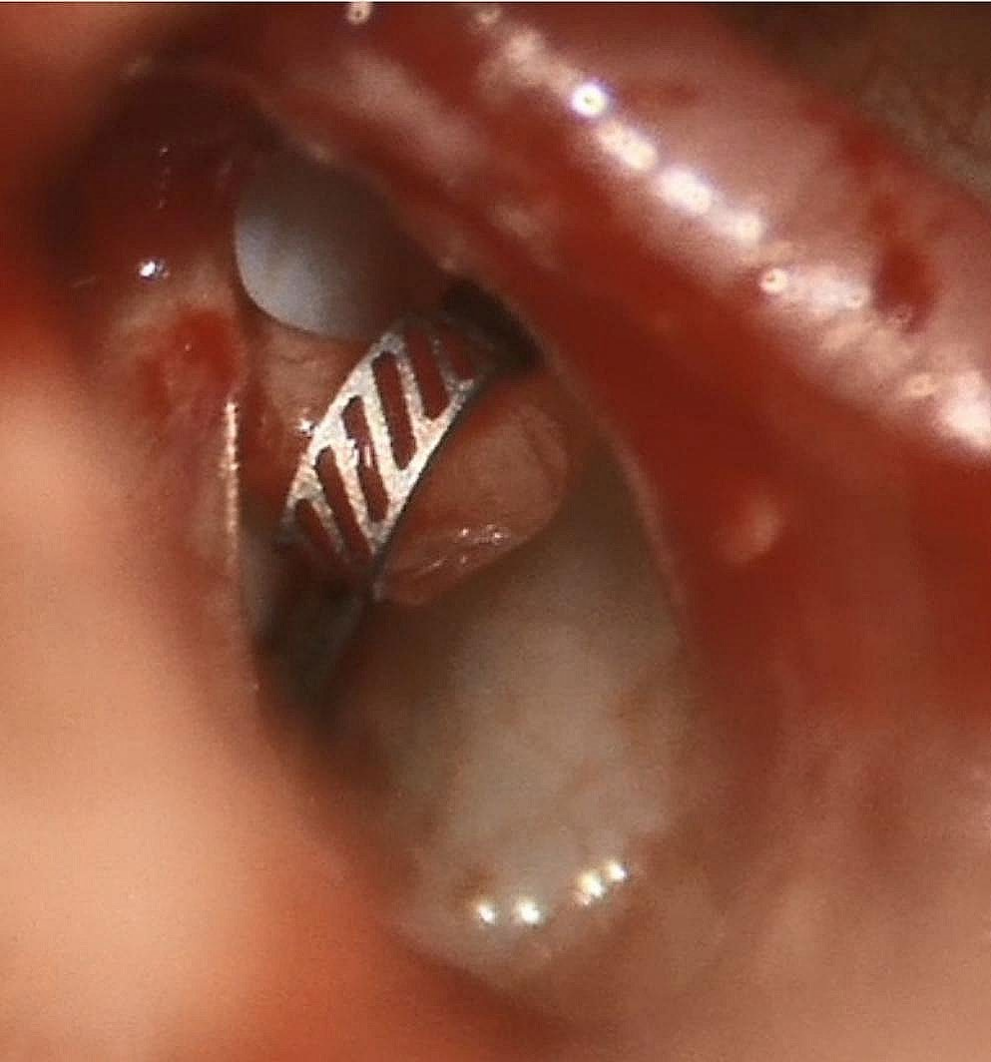
Intraoperative microscopy of the mAXIS Stapes Prosthesis before securing it onto the long process of the incus

The operations were performed under general anesthesia by two experienced ear surgeons. Access to the oval window was created through an endaural approach. The stapes crura and footplate were perforated using either a CO₂ laser with scanner (AcuPulse™ DUO + SurgiTouch™, Lumenis Germany GmbH, Hanauer Landstraße 291 A, D-60,314 Frankfurt am Main, Germany) with the “one-shot” technique [14] or diode laser (FOX 980, A.R.C. Laser GmbH, Bessemerstr. 14, 90,411 Nuernberg). The correct prosthesis length was calculated by measuring the distance between the footplate and the medial side of the long process of the incus using a stapes measuring device and adding 0.5 mm insertion depth into the perilymphatic space. After positioning the prosthesis into the perforated footplate, the loop was secured to the long process of the incus using a crimping forceps with a retaining spring with within the handle [15] (Fig. 2).

Statistical analyses were performed using GraphPad Prism (Ver. 9.1.1.). For the evaluation of normal distribution in the audiological assessment data, we applied D’Agostino & Pearson test, a statistical method that assesses normality based on skewness and kurtosis. Furthermore, we conducted a one-way repeated measures ANOVA to assess differences in air-bone gap measurements across time points. This research was carried out in Germany in accordance with the principles outlined in the Declaration of Helsinki. The study received approval from the ethics committee at the University of Goettingen (Goettingen: 1/9/20).

## Results

From January 2021 to November 2023, 30 patients underwent 34 otosclerosis surgeries involving the implantation of the mAXIS Stapes Prosthesis. All cases underwent audiometric testing before implantation. Out of these 34 cases (age: 48.7 ± 11.5), 67.6% were female (age: 50.5 ± 11.9) and 32.4% male (age: 45.1 ± 9.6).

In the majority of cases, a prosthesis length of 4.50 mm was used (*n* = 30), as well as 4.25 mm (*n* = 2) and 4.75 mm (*n* = 2). The average ST-follow-up time was at 25.1 ± 15.3 days (*n* = 34), while MT follow-up was at 181.0 ± 107.4 days (*n* = 18).

**Fig. 3:**
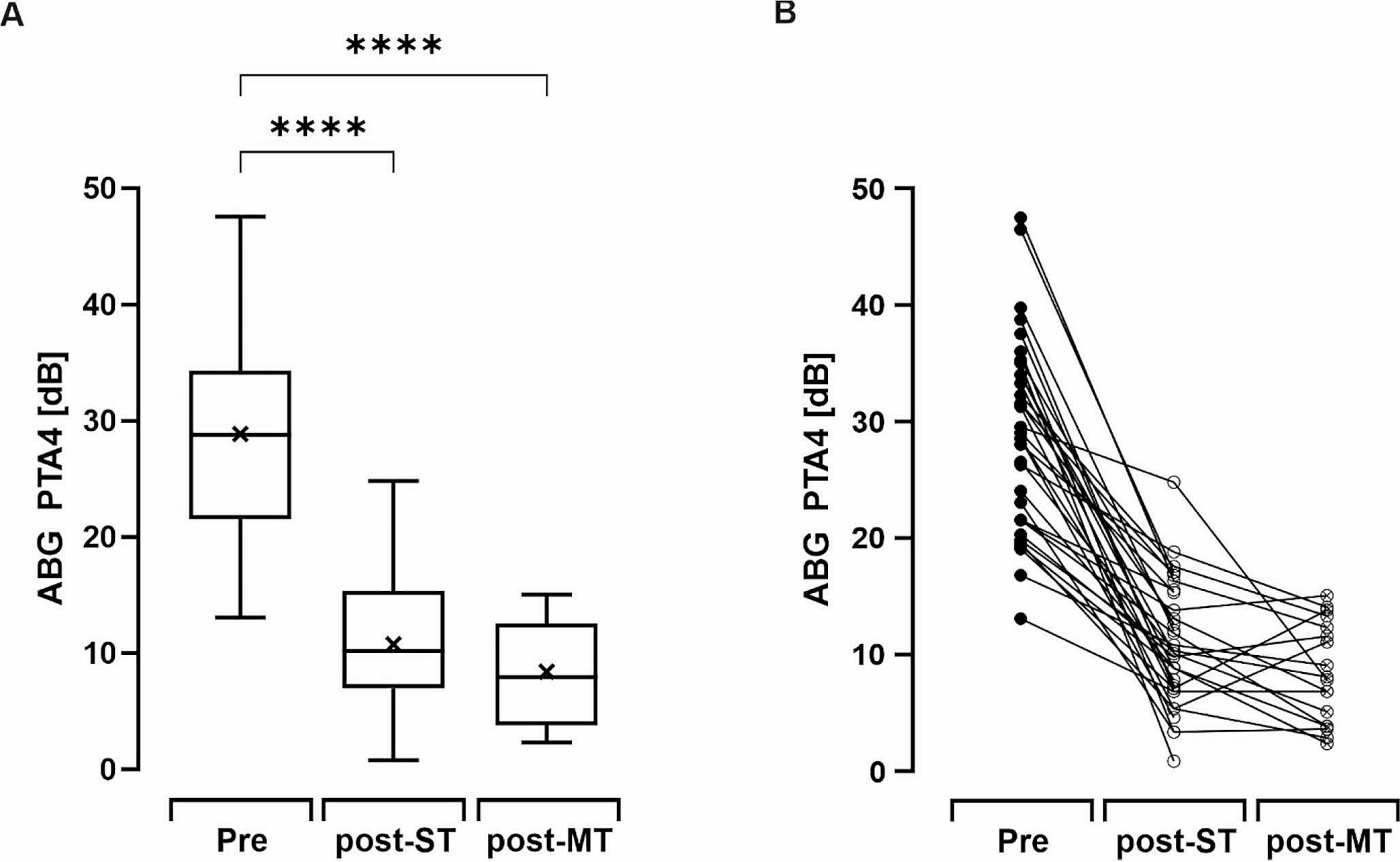
Average (**A**) and individual (**B**) PTA4 ABG (dB) measured pre- and postoperative at ST- (*n* = 34) and MT-follow-up (*n* = 18). Boxplots are shown with upper and lower limit (whiskers), median (horizontal line) and mean (cross). Statistical analysis was performed by one-way ANOVA with Turkey´s multiple comparison. Between pre to post-ST and pre to post-MT there was a significant improvement in ABG (both p-value < 0.0001), while there was no significant difference between ST and MT (p-value: 0.43)

**Fig. 4:**
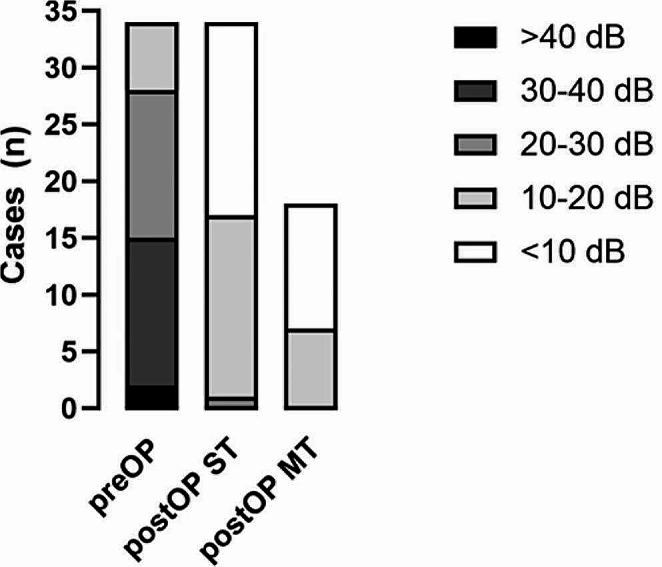
Individual pre- and postoperative PTA4 ABG. ABG-closure was within 10 dB in 50% of cases at ST follow-up (*n* = 34) and 61% at MT follow-up (*n* = 18). An ABG-closure within 20 dB was achieved by 97% of cases at ST follow-up and 100% at MT follow-up

The PTA4 ABG significantly improved from 28.8 ± 8.1 dB to 10.7 ± 5.2 dB at ST follow-up (*n* = 34) at 25.1 (± 15.3) days. The MT follow-up showed a further decrease in ABG at 8.3 ± 4.1 dB (*n* = 18; *p* = 0.43) at 181.0 (± 107.4) days. In 50% of cases at ST follow-up and 61% at MT follow-up, ABG-closure was within 10 dB. An ABG-closure within 20 dB was achieved by 97% of cases at ST follow-up and 100% at MT follow-up. (Figs. 3 and 4)

**Fig. 5:**
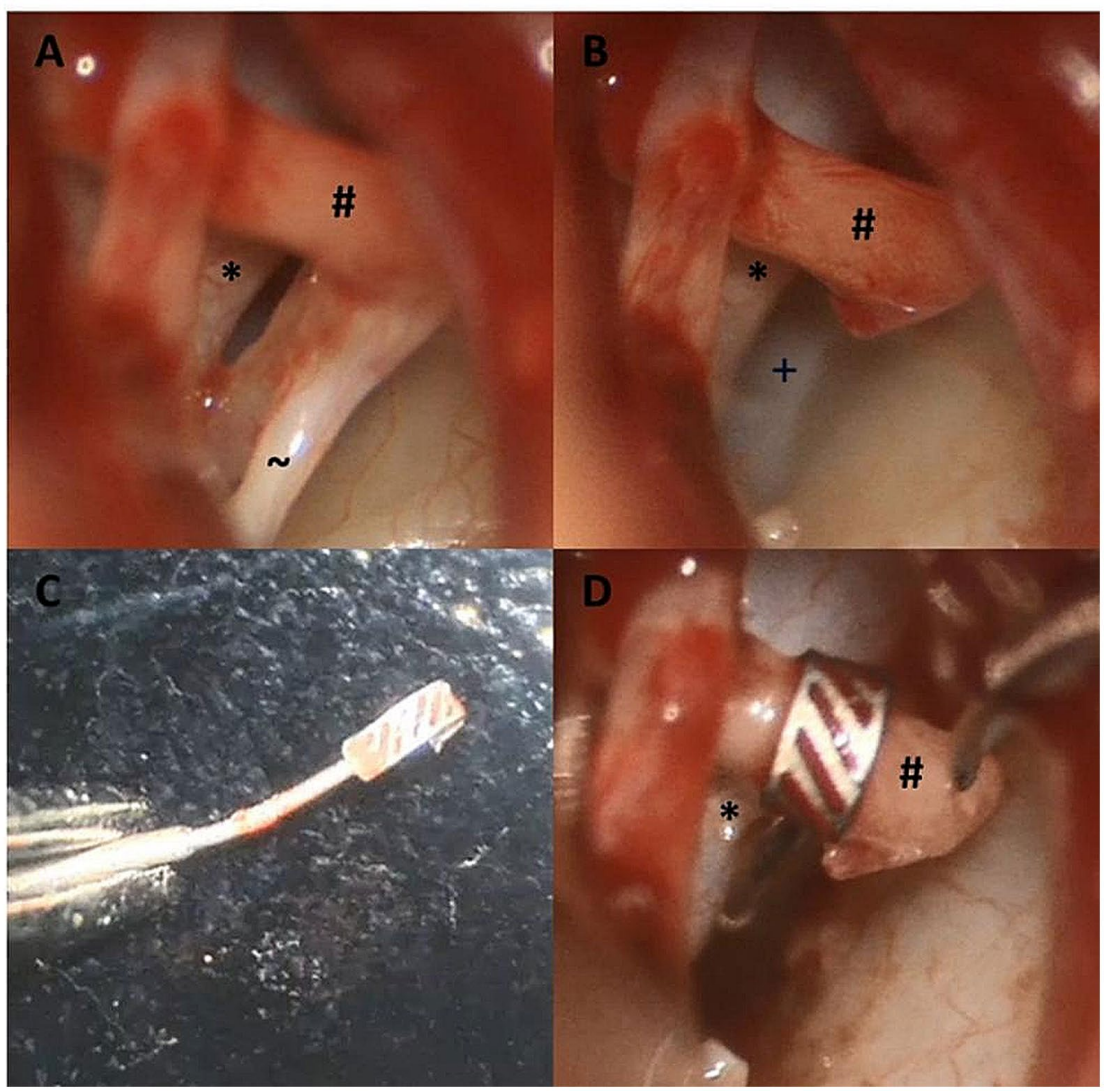
One case showed a prolapsed facial nerve (*), hindering the application of the stapes prosthesis. **A** shows intraoperative sight before the removal of the stapes suprastructure with the tendon of the stapedius muscle (∼), **B** after removal of the stapes suprastructure, showing the oval window (+) partially visible. **C** displays the bend prosthesis and **D** the intraoperative situation after securing the prosthesis to the incus (#)

The implantation of the prosthesis was straightforward and no intraoperative complications were reported. Perforation of the stapes footplate was performed using either a CO2 - or a diode laser. One revision surgery was included. One case showed a prolapsed facial nerve over the round window. Thus, conventional application of the stapes prosthesis was not possible. To circumvent this problem, the device was bend using a surgical forceps. (Fig. 5)

The normal distribution for the dataset was assessed using the D’Agostino & Pearson test. The results of the test indicated that the data exhibited a normal distribution (p-value pre: 0.73 *n* = 34; p-value ST: 0.48 *n* = 34; p-value MT: 0.09 *n* = 18).

There was no alteration in PTA4 when comparing preoperative and postoperative bone conduction results (*n* = 34). The bone conduction threshold remained consistent, transitioning from 26.4 dB HL before surgery to 26.2 dB HL postoperatively at the ST follow-up (*p* = 0.98). In two cases, a temporary decrease in bone conduction was reported. Both patients received oral corticosteroids and fully recovered within 40 days. Another case developed vertigo after 7 days, received oral corticosteroids and showed normal vestibular function 2 months after surgery, and one patient exhibited an irritating fullness of the ear leading to a revision surgery, after the patient noted a subjective improvement. None of the patients complained about tinnitus, facial nerve palsy, or taste disturbance. Throughout all follow-up assessments, the otoscopic examinations showed regular postoperative tympanic membranes.

## Discussion

Stapes surgery has proven to be an effective treatment for restoring hearing in otosclerosis patients. Throughout the years, various changes have been made to the surgical procedure and prostheses to improve intraoperative handling, coupling and postoperative audiologic outcomes. Over decades, more than 100 different stapes prosthesis have been developed [16].

This is the first report on the safety, effectiveness and handling on a new stapes prosthesis. Hearing improved in each of the 34 cases, the average PTA4 ABG decreased to 10.7 ± 5.2 dB at ST follow-up at 25 days (*n* = 34) and 8.3 ± 4.1 dB at MT follow-up at 181 days (*n* = 18; PreOP vs. ST/MT: *p* < 0.0001). In 61% of cases, ABG-closure was within 10 dB and 100% within 20 dB at MT follow-up. As the follow-up time of our patient group is heterogenous, a comparison with other literature is difficult, especially for ST follow-up. Huber et al. [17, 18] found a significant improvement of the PTA4 ABG at short- to mid-term follow-up after one year. The ABG-closure during our follow-up time of 181 days is comparable to those of other studies. Grolman et al. [11] reported an ABG-closure within 10 dB in 56.6% of cases while Sergi et al. [19] had an ABG-closure within 10 dB in 61.5% of cass, both studies reaching an ABG-closure within 20 dB in 100% of cass. The success of an otosclerosis operation is not only defined by the immediate hearing gain, but should be observed over a longer period, as the hearing outcome continues to change [20].

Individual anatomical conditions in patients may require the surgical procedure to be adapted. A prolapsed facial nerve caused difficulties in one case. Thanks to the flexibility of the thin shaft of the prosthesis, it can be individually adapted to the patient’s needs. In our case, contact with the facial nerve was prevented with the prosthesis.

The effects of different crimping methods on hearing after stapes surgery have been the subject of numerous studies. In addition to the heat activation of nitinol-prostheses, clipping, crimping and non-crimping prosthesis (such as a bucket-prosthesis) are commonly used in stapes surgery. Many favor the use of heat-crimped prostheses over manually crimped prostheses, as these are easier to handle [12]. Nevertheless, each crimping method has its own disadvantages. It is vital for hearing outcome to mitigate mucosal damage during surgery [21]. The heat-activation of nitinol-prostheses may cause vaporization of blood vessels supplying the long process of the incus [22]. However, manual crimping may cause mechanical trauma and incus erosion due to overcrimping [8, 10]. While none of the crimping methods appears to lead to an increase in adverse events, a minority of studies suggest significantly better hearing outcome with nitinol-prostheses [23–25]. It is tempting to speculate that nitinol prostheses are superior, as there are many biased studies. In addition, the cost-benefit ratio plays an important role in the choice of many surgeons, which means that many surgeons still prefer clipping or crimping prostheses to nitinol prostheses. Ultimately, the surgeon decides on the crimping method, although heat-crimped prostheses are easier to handle, especially for unexperienced stapes surgeons [26].

A stable anchorage is crucial for the hearing outcome [25] but also the length plays an important role [27]. The length of the prosthesis is individually fitted by measuring the distance between the incus and the footplate using specialized instruments [28] and remains a critical factor for hearing success [27]. While the mAXIS Stapes Prosthesis is available from 3.50 to 5.50 mm, the majority of cases in our study received a 4.50 mm prosthesis (*n* = 30), in addition to 4.25 mm (*n* = 2) and 4.75 mm (*n* = 2). Recent developments in stapes prosthesis attempted to improve the stability of anchoring and protect the mucosa of incus while giving flexibility to the surgeon [29, 30].

Extensive research was conducted on the influence of the diameter of the stapes prosthesis. Sizes range from 0.3 mm to 0.8 mm, while the most common diameter is 0.4–0.6 mm. In a metaanalysis assessing prosthesis diameter, the success rate, indicating the rate of ABG closure within 10 dB, was 67% for a diameter of 0.6 mm compared to 58% for a diameter of 0.4 mm (*p* = 0.05) [31]. As only five studies could be included in this Meta-Analysis, the authors then analyzed pooled uncontrolled data from 62 studies with 9536 cases and found a significantly better postoperative success rate with a 0.6 mm diameter compared to a 0.4 mm diameter. Postoperative BC was comparable in both groups indicating no significant risk for inner ear hearing loss by employing the 0.6 mm prosthesis. This is in line with experimental studies that suggest better results with bigger prosthesis diameters [32–34]. However, another review could not reproduce these findings [12]. All our patients were treated with a small perforation stapedotomy. The smaller the inner ear opening, the lower the risk of damage to the inner ear, hence the smaller prosthesis diameter.

While no prosthesis related adverse events were reported during the follow-up period, long-term monitoring is still required for patients. Nevertheless, the results of this study validate the audiological effectiveness and safety of the prosthesis, paving the way for surgical implantation in a larger patient population in the future.

## Conclusion

The new mAXIS Stapes Prosthesis has proven to be safe and efficient for implantation in patients suffering from otosclerosis. Future research will further delineate its long-term efficacy and safety profile.
